# Relationship Between Plasma Vitamin C and COVID-19 Susceptibility and Severity: A Two-Sample Mendelian Randomization Study

**DOI:** 10.3389/fmed.2022.844228

**Published:** 2022-03-09

**Authors:** Song Chen, Changhua Zheng, Tianlai Chen, Dianhua Huang, Yuancheng Pan, Shunyou Chen

**Affiliations:** ^1^Department of Orthopedics, Fuzhou Second Hospital of Xiamen University, School of Medicine, Xiamen University, Xiamen, China; ^2^Department of Cardiology, Fujian Medical University Union Hospital, Fuzhou, China; ^3^The Third Department of Clinical Medicine, Fujian Medical University, Fuzhou, China

**Keywords:** Vitamin C, COVID-19, Mendelian randomization study, cause effect, SNP

## Abstract

**Background:**

Considering the antioxidant function of Vitamin C, also called ascorbic acid, it is widely used against viral infections such as coronavirus disease (COVID-19) based on *in vitro*, observational, and ecological studies. Many confounding factors that can affect Vitamin C levels. Thus, the association described to date may not be causal. To determine the causal relationship between genetically predicted plasma Vitamin C and COVID-19 susceptibility and severity, we performed two-sample Mendelian randomization (MR) based on large samples.

**Methods:**

The summary-level data for Vitamin C was obtained from a GWAS meta-analysis, which included 52,018 individuals from four studies of European ancestry. Data for COVID-19 HGI results were obtained from the meta-analysis of 35 GWASs with more than 1,000,000 subjects of European ancestry, including 32,494 cases with COVID-19 susceptibility and 1,316,207 controls, 9,986 cases with COVID-19 hospitalization and 1,877,672 controls, and 5,101 cases with COVID-19 severe disease and 1,383,241 controls. Mendelian randomization (MR) analysis was conducted to examine the effect of selected single nucleotide polymorphisms and COVID-19 susceptibility, hospitalization, disease severity. Several sensitivity analyses were performed with inverse-variance weighted (random-effect model), inverse variance weighted (fixed-effect model), weighted median, and maximum likelihood methods for estimating the causal effects.

**Results:**

In this MR study, genetic predisposition to the levels of plasma Vitamin C was not associated with COVID-19 susceptibility (OR: 0.99, 95% CI: 0.84–1.17, *P* = 0.91), hospitalization (OR: 1.10, 95% CI: 0.71–1.71, *P* = 0.67) and severity (OR: 0.83, 95% CI: 0.43–1.59, *P* = 0.58). The association was consistent in complementary analyses. No potential heterogeneities and directional pleiotropies were observed for the analysis results.

**Conclusion:**

According to our study, no correlation was observed between plasma Vitamin C levels and COVID-19 susceptibility and severity. Further studies in different ethnics are necessary to explore the potential role and mechanisms of circulating serum Vitamin C levels on COVID-19.

## Introduction

Coronavirus disease (COVID-19) is characterized by cytokine storms that result in immunogenic damage, especially to the endothelium and alveolar membrane (1, 2). Globally, 267,865,289 confirmed cases of COVID-19, including 5, 285,888 deaths were recorded as of December 10, 2021 (3). Cytopathic effects initially induced by viruses followed by a cytokine storm may carry substantial health risks (4). Infected people always experience mild infections, manifested by fever and dry cough, and they usually do not require hospitalization. Some people develop severe illness, which eventually turns into ARDS (3). Vitamin C, otherwise known as ascorbic acid, is a an essential nutrient. It boosts immunity and acts as a potent antioxidant (5), promote the synthesis of vasopressin and cortisol, enhance the neutrophil extracellular trap (NET) function, and improve the body's resistance to viruses (6, 7). COVID-19 patients undergoing Vitamin C treatment showed a significant decrease in inflammatory markers, such as D-dimer and ferritin, suggesting that Vitamin C may be useful for the treatment of moderate-to-severe COVID-19 disease (6). However, two large-scaled randomized controlled trials (RCTs) have indicated that Vitamin C could not prevent sepsis and ARDS (8, 9). Besides, a meta-analysis showed that the use of vitamin C was not associated with the decreased risk of COVID-19 (10). Notably, these studies included limited sample sizes with potential confounders.

The confirmation of causal association is as challenging as the reverse causation and confounding between Vitamin C and the risk of COVID-19 susceptibility and severity. Mendelian randomization (MR) has emerged as a powerful method for identifying the causation between exposures and diseases by using genetic variants as instrument variables (IVs), which could eliminate possible confounding factors (11). Furthermore, the risk of reverse causation is also minimized, because the disease occurrence cannot affect individuals' genotypes, and single nucleotide polymorphisms (SNPs) are randomly assigned (12).

In the present study, we performed a two-sample MR to explore whether genetic evidence of individual Vitamin C traits was significantly associated with COVID19 susceptibility and severity risks.

## Methods

### Data Resources

The summary-level data for Vitamin C were obtained from a GWAS meta-analysis, which included 52,018 individuals from four studies of European ancestry (European Prospective Investigation into Cancer and Nutrition (EPIC)-CVD study (*n* = 7,650) (13), Fenland study (*n* = 10,771) (14), EPIC Norfolk study (15) (*n* = 16,756) (16), and EPIC-InterAct study (*n* = 16,841) (17), and the overlapping individuals were excluded. In EPIC-Interact and EPIC-CVD study, high-performance liquid chromatography and ultraviolet detection were used to measure plasma Vitamin C. The details are presented in **Table 2**.

The latest summary statistics data of COVID-19 were obtained from the COVID19-hg GWAS meta-analyses round 5 released publicly on January 18, 2021 (18), and the data were divided into three categories, namely, COVID-19 susceptibility, hospitalization, and severe disease outcomes (19). Documentation on the COVID-19 HGI identified the three outcome phenotypes as C2 (COVID-19 patients vs. population which were defined as any individuals who never had COVID-19), B2 (hospitalized patients with COVID-19, vs. any individuals not experiencing a hospitalization for COVID-19 including those without COVID-19), and A2 (hospitalized individuals with COVID-19 who died or required respiratory support vs. individuals without severe COVID-19 including those without COVID-19). Support for the respiratory system is characterized by intubation-ventilator-assisted breathing or high-flow nasal cannulas.

Data for COVID-19 HGI results were obtained from the meta-analysis of 35 GWASs with more than 1,000,000 subjects of European ancestry, including 32,494 cases with COVID-19 susceptibility and 1,316,207 controls, 9,986 cases with COVID-19 hospitalization and 1,877,672 controls, and 5,101 cases with COVID-19 severe disease and 1,383,241 controls.

### Selection of Genetic Instrumental Variables

All instrumental variables were associated with the Vitamin C at a genome-wide significance levels (*P* < 5 × 10^−8^) and linkage disequilibrium (LD) *r*^2^ < 0.001 at a 10,000 kb window, which confirmed the independence for the selected genetic variants (20). Then, the SNPs were extracted, which were associated with any potential confounders of the outcomes. In the present study, BMI (21) and smoking (22) were identified as confounding factors when COVID-19 was identified as the outcome (http://www.phenoscanner.medschl.cam.ac.uk/). SNP harmonization was conducted to correct the orientation of the alleles. In order to determine whether instrumental variables were weak, the F statistics were used. It was proven in MR studies that *F* > 10 can be used with strong genetic instruments.

### Statistical Analysis

MR estimate was obtained by performing an inverse variance weighted (IVW) meta-analysis of each Wald ratio. The Cochran *Q*-test was applied to check SNPs' statistical heterogeneity by using MR Egger and IVWs estimates, with *P* < 0.05 deemed significantly heterogeneous. Therefore, the IVW method based on random effects was adopted. Complementary analyses, including the inverse variance weighted (fixed effects) (23), weighted median (24), and maximum likelihood (25) methods, an outlier test Radial plot and Radial regression (26), and MR-PRESSO (27) were utilized as supplements to IVW. MR-Egger is a weighted regression approach that introduces an intercept to accommodate pleiotropy. Horizontal pleiotropy was observed when the intercept term was away from zero (28). By using this approach, unbiased estimates were achieved in the presence of pleiotropic instruments assuming that the magnitude of pleiotropic effects cannot be predicted by the size of the instrumental variables–SNPs related to plasma Vitamin C (28).

Subsequently, each SNP was excluded in a leave-one-out analysis, which allowed us to test whether individual SNPs contributed to causal associations. Pleiotropy was also assessed using funnel plots. No directional pleiotropy was observed if the funnel plot is symmetric (29).

All analyses were conducted using the “TwoSampleMR,” “MRPRESSO,” and “RadialMR” R packages in RStudio version 3.6.3. Bonferroni adjustment was used to correct for multiple comparisons (*P*-value: 0.05/3 outcomes = 0.0167). We computed two-side *P*-values, with *P* < 0.0167 regarded as statistically significant.

## Results

### Choice of Vitamin C Genetic Instruments

We extracted 647 SNPs from the GWAS meta-analysis of Vitamin C metting a genome-wide significance levels (*P* < 5 × 10^−8^). Eleven independent SNPs were associated with three COVID-19 phenotypes with linkage disequilibrium (LD) *r*^2^ < 0.001 at a 10,000 kb window, which confirmed the independence for the selected genetic variants. After harmonizing the exposure and outcomes datasets, one SNP (rs17689024) was removed for being palindromic with intermediate allele frequencies. We examined each SNP in PhenoScanner database and found that they were not significantly associated with confounding risk factors. Finally, 10 SNPs were the “Complete sets” involved in the MR analyses. The characteristics of SNPs for plasma Vitamin C are shown in Table 1. Both the exposure and outcome GWAS are summarized in Table 2.

**Table 1:** Characteristics of SNPs for plasma Vitamin C from the GWAS meta-analysis.

**Chr**	**Pos**	**SNP**	**Closet gene**	**EA**	**OA**	**EAF**	**Effect**	**SE**	***F* statistic**	***P*-value**	***N***
12	102081498	rs10128996	BCAS3	A	G	0.5925	0.0557	0.0063	78.17	<1.00E-16	52,081
17	59448945	rs1010269	BCAS3	A	G	0.1791	−0.0602	0.0082	53.90	2.56E-13	52,081
12	96249111	rs117885456	SNRPF	A	G	0.0865	0.0781	0.0116	45.33	1.69E-11	52,081
5	137517504	rs12517918	KIF20A	T	C	0.0379	−0.1107	0.017	42.40	7.61E-11	52,081
1	2326009	rs1123571	RER1	A	G	0.4477	−0.0393	0.0064	37.71	6.26E-10	52,081
5	138637444	rs11242457	MATR3	A	G	0.6912	0.0415	0.0069	36.17	2.02E-09	52,081
5	176799992	rs10051765	RGS14	T	C	0.6585	−0.039	0.0066	34.92	3.64E-09	52,081
6	52735825	rs6910581	GSTA11P	T	C	0.5693	0.0369	0.0063	34.31	4.47E-09	52,081
14	105253581	rs10136000	AKT1	A	G	0.2825	0.0404	0.0071	32.38	1.13E-08	52,081
11	61570783	rs174547	FADS1	T	C	0.6721	−0.0364	0.0066	30.42	3.84E-08	52,081

*Chr, chromosome; Pos, position for SNP; Closet gene, the nearest gene to Vitamin C associated SNP; Effect, the per-allele effect on plasma Vitamin C; P-value, the value for the genetic association; SNP, single-nucleotide polymorphism; EA, effect allele; OA, other allele; EAF, effect allele frequency; SE, standard error; N, sample size*.

**Table 2:** Sources of data for the analysis.

**Phenotype**	**Source of genetic variants**	
	**Cohort**	**Participants**
Plasma vitamin C levels	Zheng et al. (13)	• Meta-analysis of GWAS of plasma Vitamin C levels • 10,771 individuals from the Fenland study • 16,841 individuals from European Prospective Investigation into Cancer and Nutrition (EPIC)-InterAct study • 16,756 individuals from EPIC Norfolk study • 7,650 individuals from the EPIC-CVD study
COVID-19 susceptibility	Susceptibility	• Meta-analysis of 35 GWASs performed in individuals of European ancestry from 14 countries: • Cases: 32,494 individuals with COVID-19 with laboratory confirmation chart review or self-report • Controls: 1,316,207 individuals without confirmation or history of COVID-19
COVID-19 severity	Hospitalized	• Meta-analysis of 22 GWASs performed in individuals of European ancestry from 14 countries: • Cases: 9,986 hospitalized individuals with COVID-19 • Controls: 1,877,672 individuals with COVID-19 who did not undergo hospitalization
	Severe disease	• Meta-analysis of 15 GWASs performed in individuals of European ancestry from 10 countries: • Cases: 5,101 SARS-CoV-2-infected hospitalized individuals who died or required respiratory support (intubation, CPAP, BiPAP, continuous external negative pressure, or high-flow nasal cannula) • Controls: 1,383,241 without severe COVID-19

*COVID-19 susceptibility and severity outcomes are taken from the COVID-19 Host Genetics Initiative. See S1 Data for details on cohorts of COVID-19 susceptibility and severity phenotypes. BiPAP, bilevels positive airway pressure; CPAP, continuous positive airway pressure; GWAS, genome-wide association study*.

### Causal Relationships Between Plasma Vitamin C and COVID-19 Susceptibility

**Figure 1:**
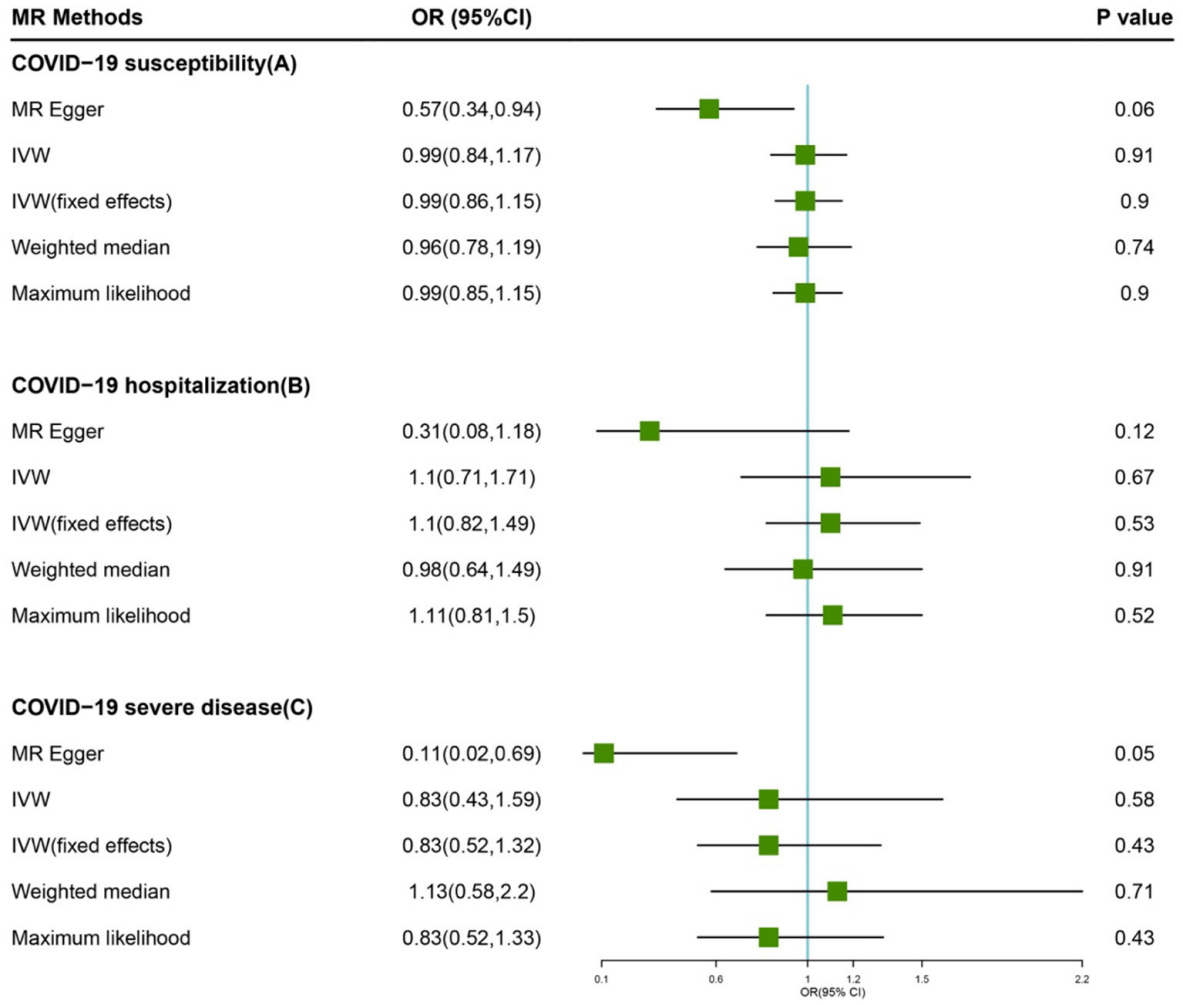
Forest plot of MR study using genetic instruments with COVID-19. **(A)** COVID-19 susceptibility, **(B)** COVID-19 hospitalization, **(C)** COVID-19 severe diseas. OR, odds ratio; IVW, inverse variance weighted; CI, confidence interval; MR, Mendelian randomization.

**Figure 2:**
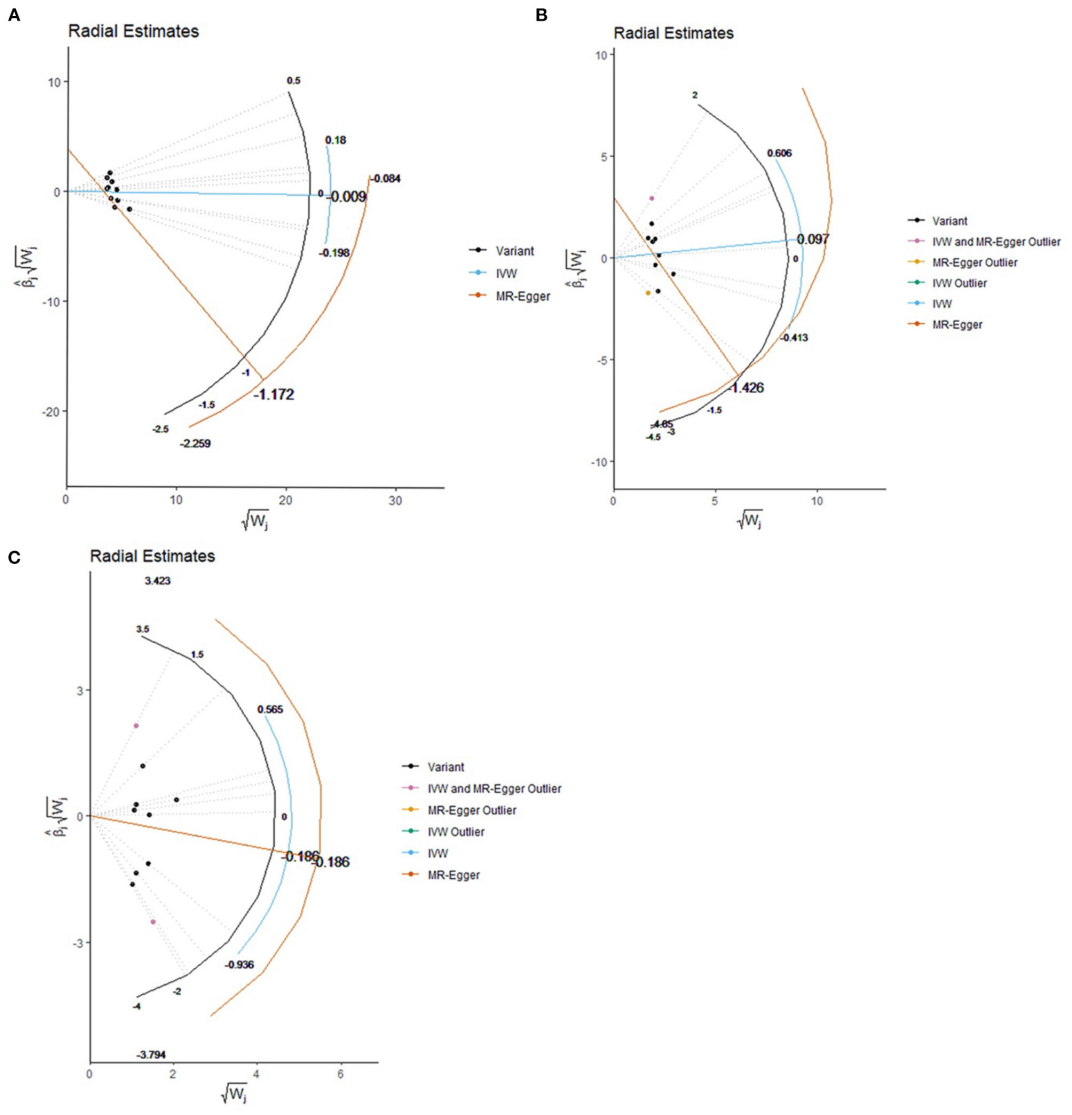
Effect estimate for each individual variant via the radial plot and radial regression. **(A)** COVID-19 susceptibility, **(B)** COVID-19 hospitalization, **(C)** COVID-19 severe disease. IVW, inverse variance weighted.

Based on IVW analyses, no causal relationship was found between plasma Vitamin C and COVID-19 susceptibility (OR: 0.99, 95% CI: 0.84–1.17, *P* = 0.91; Figure 1A). No potential heterogeneities and directional pleiotropies were observed for the analysis results (Supplementary Table 1). The radial inverse variance weighted (IVW) and MR-Egger model were employed using a range of weighting specifications, indicating that no outliers were determined with respect to their contribution to global heterogeneity (Figure 2A). Moreover, MR PRESSO analysis did not reveal any outliers. MR-PRESSO global test resulted in a *P*-value of 0.31, indicating the absence of significant heterogeneity. The forest plot is shown in Supplementary Figure 1, and the leave-one-out plot is shown in Supplementary Figure 2.

### Causal Relationships Between Plasma Vitamin C and COVID-19 Hospitalization

The random-model IVW estimate showed that genetically predicted Vitamin C levels were not significantly associated with COVID-19 hospitalization risks (OR: 1.10, 95% CI: 0.71–1.71, *P*-value = 0.67; Figure 1B). The association was consistent with complementary analyses by using MR-Egger, IVW (fixed effects), weighted median methods, and maximum likelihood methods (Figure 2). Potential heterogeneities were present, but directional pleiotropies were absent for the analysis results (Supplementary Table 1). Both radial IVW and radial MR-Egger model indicated the absence of outliers (Figure 2B). Based on MR PRESSO analysis, we found an SNP (rs11242457) that was a potential source of heterogeneity (Supplementary Figure 3). After excluding this SNP, the results of the five MR methods suggested no causal association between plasma Vitamin C and COVID-19 hospitalization. No heterogeneity was found based on the Cochran's Q statistic [*Q*value (df) = 7.67 (7), *P* = 0.36 for MR Egger method; *Q-*value (df) = 11.61 (8), *P* = 0.17 for IVW method]. The forest plot is shown in Supplementary Figure 3, and the leave-one-out plot is shown in Supplementary Figure 4.

### Causal Relationships Between Plasma Vitamin C and COVID-19 Severe Disease

First, we carried out a meta-analysis of the effects of the genetic instruments by using IVW. Increasing plasma Vitamin C levels by one standard deviation, and no significant effect was observed on susceptibility odds (OR: 0.83, 95% CI: 0.43–1.59, *P*-value = 0.58; Figure 1C). No potential heterogeneities and directional pleiotropies were observed in the analysis results (Supplementary Table 1). Outliers were detected in radial IVW and MR-Egger model (Figure 2C). However, no outlines were observed. Detailed forest plot is shown in Supplementary Figure 5. The leave-one-out analysis in Supplementary Figure 6 showed that none of the single SNP substantially affected the overall risk estimation.

## Discussion

Limited MR studies have focused on Vitamin C and COVID-19 susceptibility and severity (30). The sample size, selection and elimination of SNPs, choice of MR method of our study are all different compared with it, but the final conclusions are similar. This also further confirms that there is no significant causal relationship between vitamin C and COVID19. In terms of the MR study about Vitamin C and COVID-19, our research is the largest MR study to date. Our findings found no evidence that the genetic markers of plasma vitamin C are related to COVID-19 susceptibility and severity, and these results are inconsistent with those reported in observational studies. Considering that the confounding effects of factors can be difficult to control even with advanced statistical adjustments, such as socioeconomic, institutionalization, dietary, medical comorbidities, and lifestyle behavioral factors, associations between Vitamin C and COVID-19 may not be clear (31).

The pro-inflammatory state caused by the SARS-CoV-2 virus is the main pathophysiological process of the COVID-19, which is characterized by increased levels of serum interleukin-1,6 (IL-1,6) and tumor necrosis factor (TNF), resulting in “cytokine storm” and ARDS (32, 33). Various Vitamins, anti-oxidants, and immunomodulators have been investigated to curtail inflammatory chain reaction. In addition to its anti-inflammatory properties, Vitamin C contains free radial oxygen and nitrogen-caging properties (34). In a prospective study involving 19,357 people, after more than 20 years of follow-up, results show that people with baseline plasma Vitamin C levels in the top quartile had a 30% reduction in the risk of pneumonia (35). In experimental animal models, by inhibiting neuronal nitric oxide synthase-derived NO, Vitamin C injection into septic mice prevented impaired vasoconstriction, this promoting the dissolution of capillary microthrombi (36). These observational studies cannot eliminate the influence of confounding factors and reverse causality. In a randomized clinical trial that included 216 septic shock patients, treatment with intravenous Vitamin C, thiamine, and hydrocortisone did not significantly prolong the duration of time alive and free of vasopressor administration over 7 days, compared with intravenous hydrocortisone alone (9). Based on a meta-analysis of Vitamin C and COVID-19 susceptibility and severity including six RCTs (*n* = 572 patients), Vitamin C did not reduce mortality, ICU stays, hospital stays, and the need for invasive mechanical ventilation with high Vitamin C therapy (10). In a separate sub-group analysis, no observable benefit could be seen for severe vs. non-severe illness, or the route of administration (IV or oral) (10). Accordingly, findings from the largest randomized trial conducted to date are in accordance with those of our MR.

Our analysis has several strengths. We utilized the largest cohort of COVID-19 cases available to date, and this study involved the largest research study on genetic determinants of Vitamin C levels, which could overcome the limitations of conventional epidemiological study designs, such as confounding and reverse causality. This study is more time-efficient and less expensive than RCT. This study has some limitations. First, our datasets included the European populations, which limited the applicability of results to non-European populations. However, similar results have been obtained in populations with different ethnicities in previous randomized controlled trials of vitamin C supplementation (37). Moreover, MR's linear effect assumption could not be used to further investigate non-linear causality (38). In addition, a canalization effect could not be ruled out by our study (i.e., dilution of the gene-exposure association). Thus, the estimate might be inflated. Finally, the directional pleiotropy cannot be excluded, which is almost completely mediated through other causal pathways.

## Conclusion

The results of the present study showed no correlation between plasma Vitamin C levels and COVID-19 susceptibility and severity. Further studies in different ethnics are necessary to explore the potential role and mechanisms of circulating serum Vitamin C levels on COVID-19.

## Data Availability Statement

The original contributions presented in the study are included in the article/Supplementary Material, further inquiries can be directed to the corresponding author/s.

## Author Contributions

SoC and TC participated in study design. SoC, DH, and YP acquired and analyzed the data. ShC participated in the study's supervision. SoC drafted the manuscript. All authors contributed to the article and approved the submitted version.

## Funding

This study was supported by the Science and Technology Plan Project of Fuzhou Science and Technology Bureau in 2019 (Grant No. 2019-SZ-10).

## Conflict of Interest

The authors declare that the research was conducted in the absence of any commercial or financial relationships that could be construed as a potential conflict of interest.

## Publisher's Note

All claims expressed in this article are solely those of the authors and do not necessarily represent those of their affiliated organizations, or those of the publisher, the editors and the reviewers. Any product that may be evaluated in this article, or claim that may be made by its manufacturer, is not guaranteed or endorsed by the publisher.

## Abbreviations

COVID-19: Coronavirus disease
MR: Mendelian randomization
Chr: chromosome
Pos: position for SNP
SNP: single-nucleotide polymorphism
EA: effect allele
OA: other allele
EAF: effect allele frequency
SE: standard error.
