# Supplementary material for: Relationship Between Plasma Vitamin C and COVID-19 Susceptibility and Severity: A Two-Sample Mendelian Randomization Study
Source: Front Med (Lausanne). 2022 Mar 9;9:844228. doi: 10.3389/fmed.2022.844228 (PMC8959865; doi:10.3389/fmed.2022.844228)

Supplementary Material

**1 Supplementary Table**

**Table 1. *Summary of the Mendelian randomization of the causal effect of the plasma Vitamin C concentration on three COVID-19 phenotypes using different MR methods***

| **Exposure** | **Outcome** | **No. of IVs** | **MR results** | | | **Heterogeneity tests** | | | **horizontal pleiotropy test** | | |
| --- | --- | --- | --- | --- | --- | --- | --- | --- | --- | --- | --- |
|  |  |  | **Method** | **Beta** | **P** | **Methods** | **Cochran's Q (p)** | | **MR-Egger intercept(p)** | | |
|  | COVID-19 susceptibility | 10 | MR Egger | -0.56 | 0.06 | MR Egger | 6.11(0.63) | | 0.03(0.06) | | |
|  |  |  | IVW | -0.009 | 0.91 | IVW | 11.14(0.27) | |  | |  |
|  |  |  | IVW (fixed effects) | -0.009 | 0.90 |  |  | |  |  |  |
|  |  |  | Weighted median | -0.04 | 0.74 |  |  | |  |  |  |
|  |  |  | Maximum likelihood | -0.009 | 0.90 |  |  | |  |  |  |
|  |  |  |  |  |  |  |  | |  |  |  |
|  | COVID-19 hospitalization | 10 | MR Egger | -1.17 | 0.12 | MR Egger | 13.31(1.10) | | 0.06(0.09) | | |
|  |  |  | IVW | 0.10 | 0.67 | IVW | 19.58(0.02) | |  |  |  |
|  |  |  | IVW (fixed effects) | 0.10 | 0.53 |  |  | |  |  |  |
|  |  |  | Weighted median | -0.02 | 0.91 |  |  |  |  |  |  |
|  |  |  | Maximum likelihood | 0.10 | 0.52 |  |  |  |  |  |  |
|  |  |  |  |  |  |  |  |  |  |  |  |
|  | COVID-19 severe disease | 10 | MR Egger | -2.21 | 0.05 | MR Egger | 10.90(0.21) | | 0.10（0.05） | | |
|  |  |  | IVW | -0.19 | 0.58 | IVW | 17.83(0.04) | |  |  |  |
|  |  |  | IVW (fixed effects) | -0.19 | 0.43 |  |  |  |  |  |  |
|  |  |  | Weighted median | 0.13 | 0.71 |  |  |  |  |  |  |
|  |  |  | Maximum likelihood | -0.19 | 0.43 |  |  |  |  |  |  |

*IVW: inverse variance weighted;*

**2 Supplementary Figure**

**Figure 1**. The forest plot for the causal effects of plasma Vitamin C-associated SNPs on COVID-19 susceptibility


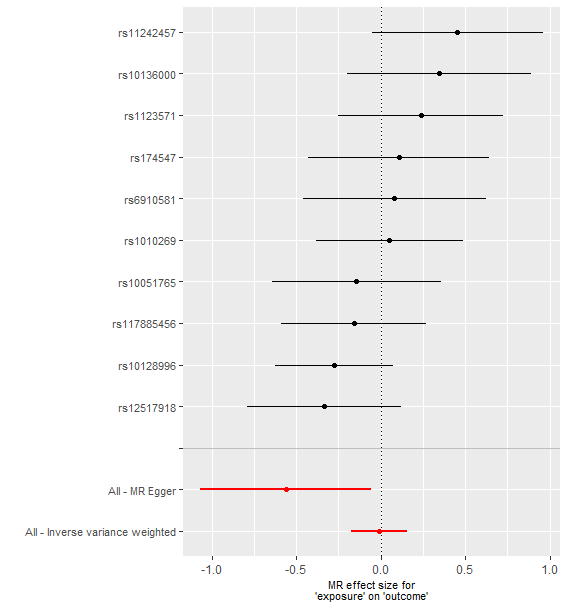


**Figure 2.** Leave-one-out sensitivity analysis for COVID-19 susceptibility using genetic instruments;


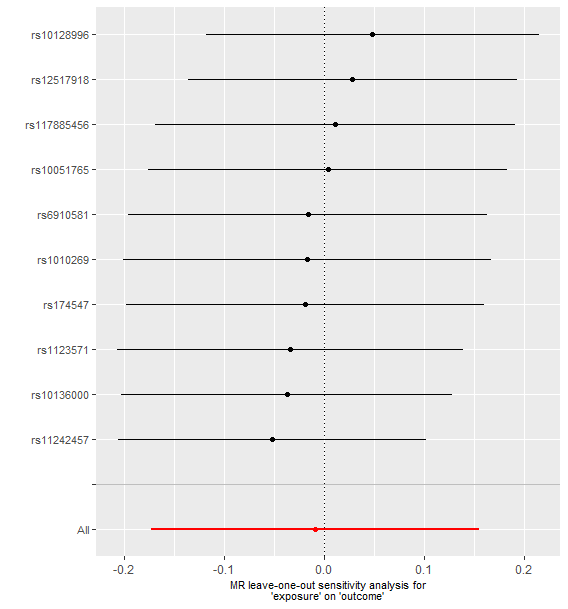


**Figure 3.** The forest plot for the causal effects of plasma Vitamin C-associated SNPs on COVID-19-hospitalization;


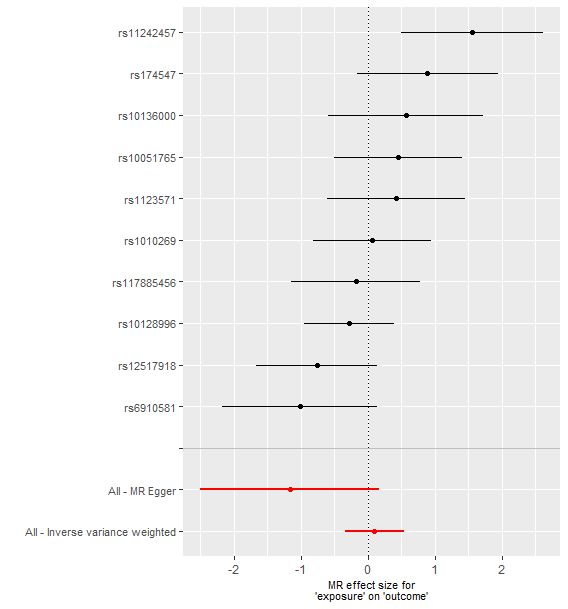


**Figure 4**. Leave-one-out sensitivity analysis for COVID-19 hospitalization;


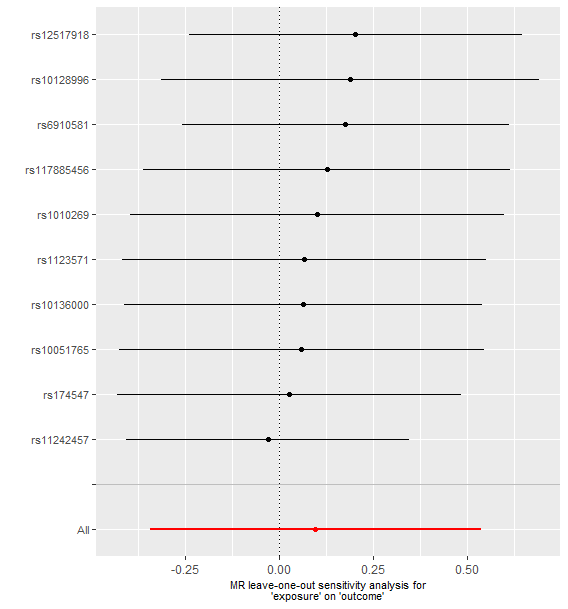


**Figure 5**. The forest plot for the causal effects of plasma Vitamin C-associated SNPs on COVID-19 severe disease;
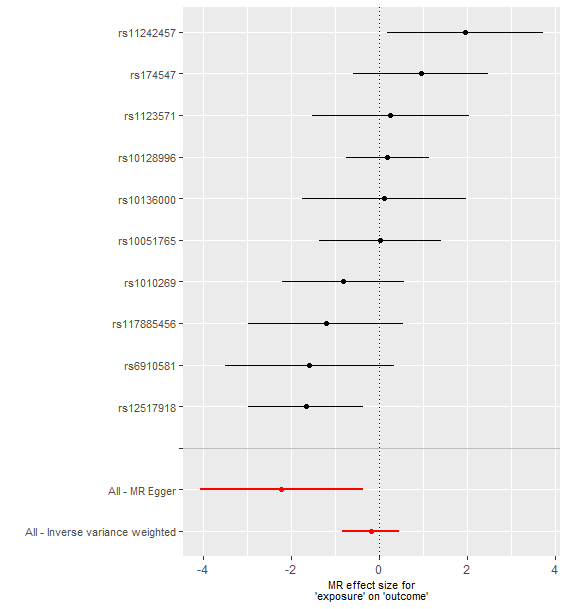


**Figure 6**. Leave-one-out sensitivity analysis for COVID-19 severe disease;
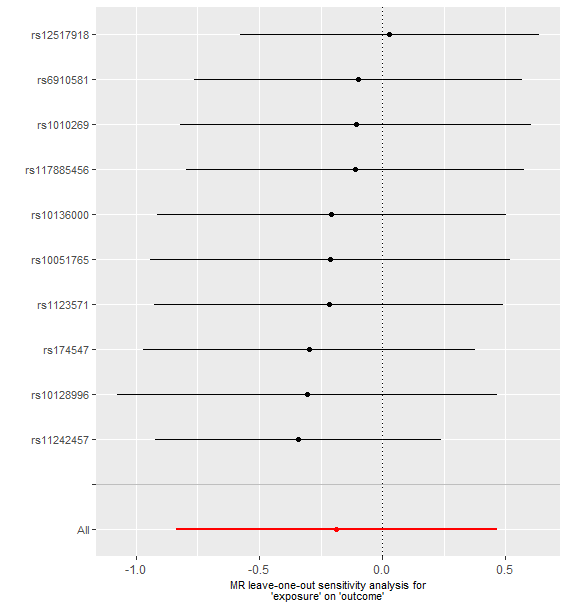

Supplement: Supplementary file 1 [file Data_Sheet_1.docx]
